# The Association Between the Concentration of Serum Magnesium and Postmenopausal Osteoporosis

**DOI:** 10.3389/fmed.2020.00381

**Published:** 2020-08-04

**Authors:** Jinlei Chang, Degang Yu, Jindou Ji, Ning Wang, Shengwen Yu, Bo Yu

**Affiliations:** ^1^The First Clinical Medical College, Shandong University of Traditional Chinese Medicine, Jinan, China; ^2^Department of Orthopaedics, Shanghai Ninth People's Hospital, School of Medicine, Shanghai Jiao Tong University, Shanghai, China; ^3^The College of Traditional Chinese Medicine, Shandong University of Traditional Chinese Medicine, Jinan, China; ^4^Department of Orthopaedics, Affiliated Hospital of Shandong University of Traditional Chinese Medicine, Jinan, China

**Keywords:** serum magnesium, postmenopausal, osteoporosis, osteopenia, meta-analysis

## Abstract

**Background:** Osteoporosis is the most common and widespread chronic skeletal metabolic disease in the world and can lead to catastrophic fractures. Therefore, it is important to look for factors that can be modified or controlled to prevent osteoporosis. Although serum Mg is believed to be associated with osteoporosis in many individuals, there are conflicting reports on the association between serum Mg and osteoporosis. Therefore, this meta-analyses aimed to explore the association between the concentration of serum Mg and osteoporosis as well as that between the concentration of serum Mg and osteopenia.

**Methods:** Articles were searched in PubMed. We also reviewed the reference lists of the relevant publications and reviews as of December 2019. Finally, 11 eligible studies involving 2,776 postmenopausal women were selected. We performed subgroup analysis, and publication bias was assessed.

**Results:** According to the forest plot analysis, postmenopausal women with osteoporosis had a lower concentration of serum Mg than normal controls [standardized mean difference (SMD) = −0.56, 95% confidence interval (CI) = −1.02 to −0.09]. However, this result was not applicable to those with osteopenia (SMD = −0.30, 95% CI = −0.69 to 0.09). The subgroup analysis by geographical location found a similar pattern in European postmenopausal women with osteoporosis (SMD = −0.73, 95% CI = −1.322 to −0.143), but not in Asian (SMD = −0.007, 95% CI = −0.381 to 0.394). The subgroup analysis by site of bone mineral density (BMD) showed the serum Mg concentration of postmenopausal women with osteoporosis (BMD of femur) was lower than in healthy controls (SMD = −0.44, 95% CI = −0.77 to −0.12), and BMD of the spine group had the same conclusion (SMD = −0.78, 95% CI = −1.36 to −0.19). Besides, the serum Mg concentration of postmenopausal women with osteoporosis was lower than that of the normal bone mass group in the studies those included more than 50 postmenopausal women with osteoporosis (SMD = −0.57, 95% CI = −1.04 to −0.11). We also found postmenopausal women under the age of 60 with osteoporosis had a lower concentration of serum Mg than the healthy controls (SMD = −0.61, 95% CI = −1.09 to −0.13).

**Conclusion:** Postmenopausal women with osteoporosis have a lower concentration of serum Mg. However, the association between the concentration of serum Mg and osteopenia needs further confirmation.

## Introduction

Osteoporosis is defined as progressive systemic skeletal disease, characterized by low bone mass and microstructure deterioration of bone tissue, reduced bone mineral density (BMD) and bone strength that increase the risk of fracture (1–4). Meanwhile, osteopenia is a chronic skeletal metabolic disorder with a BMD lower than normal but not as low as that in osteoporosis, which usually increases in severity with age and is most common in postmenopausal women (5, 6). The endpoint of osteopenia is osteoporosis. The gold standard for the diagnosis of osteoporosis is using dual-energy X-ray absorptiometry to measure BMD in the lumbar spine and hips. The value of T-score can be divided into normal bone mass (T-score ≥ −1), osteopenia (−1> T-score > −2.5) and osteoporosis (T-score ≤ −2.5). A terrible consequence of osteoporosis is primarily the osteoporotic fracture. According to the statistics, the incidence of osteoporotic fractures in postmenopausal women is 40 percent or higher in Western Europe and the remaining lifetime probability was ~12%. In addition, the annual number of fragility fractures will rise 4.5 million in 2025 in the EU (7). This means a high personal and social price to pay for osteoporosis.

In addition to the well-known risk factors (e.g., nutritional deficiency), there is increased interest in the role of trace elements and vitamin deficiencies associated with osteoporosis (8). Current recommendations regarding nutrient factors for the prevention of osteoporosis are calcium, phosphorus, vitamin D, magnesium (Mg), and fluoride. Among these factors, Mg is the least reported in the literature. Mg deficiency can lead to osteoporosis mainly through the following mechanisms: (1) changing the mechanism of hydroxyapatite affects bone mineralization, and enhancing bone turnover by stimulating the function of osteoclasts; (2) destroying the homeostasis of calcium by affecting parathyroid hormone (PTH) and 1,25(OH)2-vitamin D, which could lead to hypocalcemia; (3) promoting inflammation, by inflammatory cytokines stimulating remodeling and osteopenia; (4) promoting endothelial dysfunction (9). It has been confirmed in animal and human experiment models that Mg deficiency is associated with reduced osteoclastic and osteoblastic activity, osteopenia, and skeletal fragility (10, 11). In rat models with severely deficient Mg diets, impaired bone growth and exacerbation of loss of bone mass were observed (12–14). Micro-computed tomography revealed that the number of femoral trabecular bones are decreased, and the BMD subsided in distal metaphysis in the low Mg mice (15). *In vitro*, osteoblasts proliferate rapidly in a dilution of one-fold concentration of Mg extract, indicating that the extract of high concentration of Mg can promote the proliferation/differentiation behavior of osteoblasts (16).

Therefore, clarifying the association among the concentration of serum Mg, osteoporosis, and osteopenia can help in the formulation of clinical policy and guidelines. Therefore, this meta-analyses aimed to explore the association between the concentration of serum Mg and osteoporosis as well as that between the concentration of serum Mg and osteopenia.

## Methods

### Search Strategy

All articles that were indexed up to December 2019 and published in PubMed were searched. Literature searches were performed using free text words. The search keywords used were: “serum magnesium” OR “magnesium” AND “osteoporosis” AND “osteopenia.” Additionally, to find other publications, articles cited in the references of identified relevant articles were used. We included studies written in all languages.

### Selection Criteria

The articles were independently selected and reviewed by three authors (DY, JJ, NW) who started by screening titles and abstracts according to the relevance of the topic. After reading the abstract, the full text was screened for appropriate studies to include in the meta-analysis. Eligible studies for inclusion were independently selected by two authors (JJ, NW). When it was not clear whether a study should be included or not, there was a discussion with the third author (DY) to establish a consensus.

The inclusion criteria for eligible studies were as follows: (1) studies involving human subjects, (2) observational studies, (3) studies focusing on the association between the concentration of serum Mg and osteoporosis or osteopenia, (4) studies included data on the concentration of serum Mg for patients with osteoporosis or osteopenia and healthy individuals, and (5) studies that did not characterize diseases and drug intake that might influence the concentration of serum Mg.

The exclusion criteria were as follows: (1) studies with animal subjects, (2) *in vitro* or laboratory studies, (3) reviews or case reports, (4) studies not providing exact data on the concentration of serum Mg, (5) studies with diseases or medications that may affect the concentration of serum Mg, and (6) studies with sample size <10.

### Data Extraction and Quality Assessment

The two authors (DY, SY) used a standard form to extract data independently. The following information was extracted from each included study: the surname of the first author, year of publication, continent, the sample size, and data on the concentration of serum Mg in different groups (mean ± SD) (Table 1).

**Table 1:** Summary characteristics of studies and participants.

**Name**	**Year**	**Design**	**Location**	**Osteoporosis' number**	**The level of serum Mg (Mean** **±** **SD) mg/dl**			**Age of subjects (years)**	**BMD instrument**	**Site of BMD**
					**Osteoporotic**	**Osteopenia**	**Healthy**			
Reginster	1989	Case-control	European	10	1.968 ± 0.144		2.088 ± 0.096	≥60	Dual-photon absorptiometry	Thoracic spine, lumbar spine
Ali	2002	Case-control	European	70	2.699 ± 0.738		3.252 ± 0.623	≥60	DEA	Spine, femur
Berna	2010	Case-control	European	25	2.07 ± 0.31	2.13 ± 0.42	2.28 ± 0.63	<60	DEA	L2–L4 spine
Chen	2007	Case-control	Asia	113	1.776 ± 0.2928	1.771 ± 0.2904	1.785 ± 0.2376	≥60	DEA	L1–L4 spine
Liu	2009	Case-control	Asia	123	1.968 ± 0.252	1.912 ± 0.281	1.935 ± 0.221	<60	DEA	L1–L4 spine, Femoral neck, Ward's triangle, greater trochanter, and intertorch of femur
Mutlu	2007	Case-control	European	40	1.7 ± 0.2	2.2 ± 0.2	2.7 ± 0.4	<60	DEA	Femoral neck
Bahtiri	2015	Case-control	European	49	2.594 ± 0.2400	1.968 ± 0.2160	1.992 ± 0.2160	≥60	DEA	Total hip, Femoral neck, L1–L4 spine
Mederle	2018	Case-control	European	132	1.76 ± 0.06		2.14 ± 0.14	≥60	DEA	Lumbar spine, femoral
Wang	2006	Case-control	Asia	77	2.184 ± 0.2640	2.112 ± 0.2160	2.088 ± 0.1920	≥60	DEA	Spine, Femur neck
Razmandeh	2014	Case-control	Asia	30	1.508 ± 0.489		1.980 ± 0.823	≥60	DEA	L1–L4 spine, total femoral
Okyay	2013 (1)	Case-control	European	142	0.86 ± 0.1		0.89 ± 0.1	<60	DEA	L1–L4 spine
Okyay	2013 (2)	Case-control	European	71	0.84 ± 0.16		0.89 ± 0.17	<60	DEA	Total femoral
Okyay	2013 (3)	Case-control	European	102	0.86 ± 0.18		0.89 ± 0.16	<60	DEA	Femoral neck
Okyay	2013 (4)	Case-control	European	95	0.85 ± 0.1		0.94 ± 0.1	≥60	DEA	L1–L4 spine
Okyay	2013 (5)	Case-control	European	65	0.85 ± 0.14		0.91 ± 0.18	≥60	DEA	Total femoral
Okyay	2013 (6)	Case-control	European	87	0.86 ± 0.16		0.91 ± 0.17	≥60	DEA	Femoral neck

We used the Newcastle-Ottawa Scale to assess the qualities of all included studies and most of the included literature was rated above 7.

### Statistical Analysis

We analyzed the extracted data from the included studies with a meta-analysis and present the results with a standardized mean difference (SMD) and 95% confidence intervals (CI). Chi-square (χ^2^) and I-square (*I*^2^) tests were used to test the heterogeneity between studies. When we used the χ^2^-test, we interpreted *P* < 0.10 to mean there was significant heterogeneity. When heterogeneity was detected, we used a DerSimonian-Laird random-effects model (17). We used a Mantel-Haenszel fixed-effect model when no heterogeneity was detected.

I^2^ is believed to be a better measure of the consistency between trials in a meta-analysis, because it describes the percentage of total variation across studies that is due to heterogeneity rather than chance (18). *I*^2^ values of 25, 50, and 75% were considered to be low, medium, and high heterogeneity (19).

We performed subgroup analyses to identify associations between the concentration of serum Mg and the characteristics of studies to examine if this could explain heterogeneity. We used Begg's and Egger's regression tests and trim-and-fill funnel plots to assess publication bias (20–22).

## Results

### Literature Search

**Figure 1:**
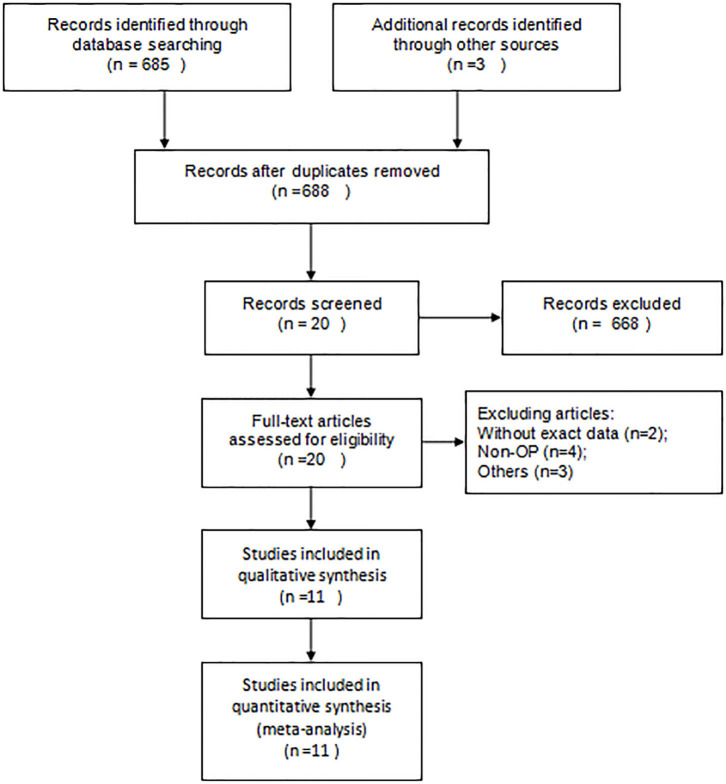
Flowchart of the studies selection.

From the initial 475 articles, 455 were excluded after reading titles and abstracts because they did not meet the inclusion criteria. Twenty articles were included for full-text assessment, from which 9 were excluded from the sample studies: two did not have data on serum Mg; four mentioned osteoporosis, but their main target was osteoporotic fractures; two involved patients administered with drugs that improved the concentration of serum Mg|; one compared the concentration of serum Mg between osteoporosis and osteopenia. We included a total of 11 eligible papers, representing 2,776 postmenopausal women from 16 case-control studies [Figure 1; (6, 8, 23–31)]. Among them, according to the age and the location of measurement, Okyay's study (26) was divided into six part. In 10 of the studies, participants were over 60 years old. Except for Reginster's study (25), other studies have measured BMD with DEA.

### Meta-Analysis Results

**Figure 2:**
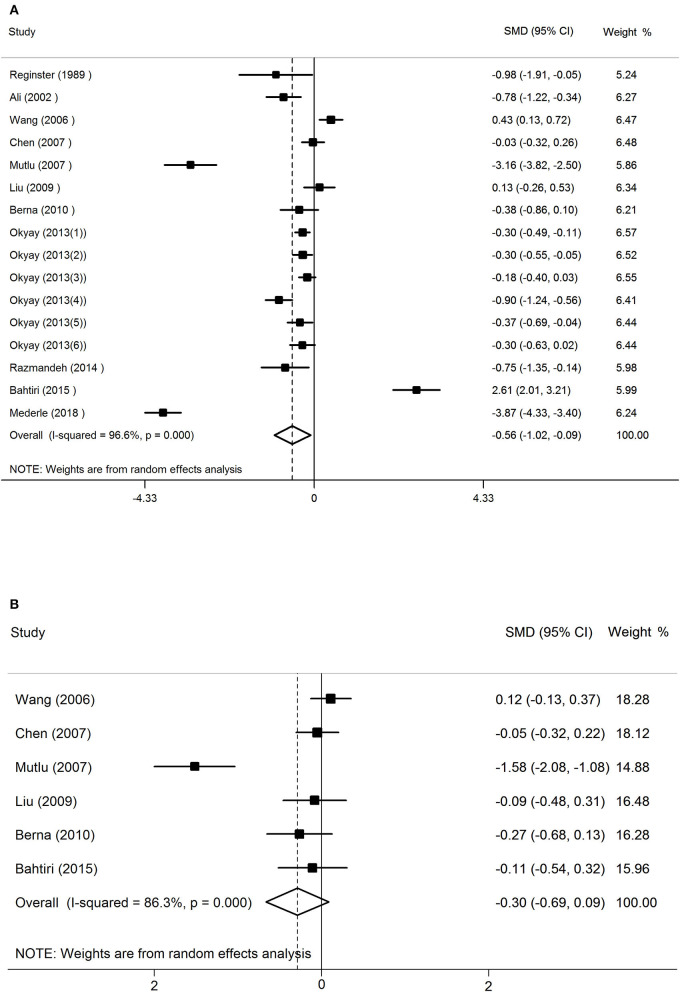
The forest plot for studies on the concentration of serum Mg in osteoporosis and the normal controls **(A)**, osteopenia vs. healthy controls **(B)**.

The results of random-effects meta-analysis showed that women with osteoporosis had a lower concentration of serum Mg than the normal controls (SMD = −0.56, 95% CI = −1.02 to −0.09). The 16 sets of results showed a statistically significant heterogeneity (*I*^2^ = 96.6%, *P* < 0.001; Figure 2).

The random-effects forest plot for studies on the concentration of serum Mg in women with osteopenia vs. healthy controls had different results (SMD = −0.30, 95% CI = −0.69 to 0.09). This meant that in postmenopausal women, the relationship between serum magnesium concentration and osteopenia is not significant. The 16 sets of results showed a statistically significant heterogeneity (*I*^2^ = 86.3%, *P* < 0.001; Figure 2).

### Results of Subgroup Analysis

The subgroup analyses showed that concentrations of serum Mg varied by geographical location. A lower concentration of serum Mg was found in European postmenopausal women with osteoporosis than in healthy controls (SMD = −0.73, 95% CI = −1.322 to −0.143), but not in Asian postmenopausal women with osteoporosis (SMD = −0.007, 95% CI = −0.381 to 0.394; Table 2).

**Table 2:** Subgroup analysis to investigate the relationship between geographical location, sites of BMD, age, osteoporosis' number, and serum Mg.

**Subgroups**	**No. of studies**	**SMD (95% CI)**	***I*^**2**^ (%)**	***P***
**Geographical location**				
Asia	4	−0.007 (−0.381 to 0.394)	97.2%	0.973
European	12	−0.73 (−1.322 to −0.143)	77.1%	0.015
**Site of BMD**				
Spine	5	−0.44 (−0.77 to −0.12)	75.6%	0.003
Femur	5	−0.78 (−1.36 to −0.19)	94.4%	0
**Age**				
≥60	10	−0.49 (−1.29 to 0.30)	97.5%	0
<60	6	−0.61 (−1.09 to −0.13)	93.6%	0
**Osteoporosis' number**				
<50	5	−0.53 (−2.33 to 1.28)	97.6%	0
≥50	11	−0.57 (−1.04 to −0.11)	96.4%	0

In addition, we also conducted subgroup analysis based on the site of BMD, the included studies were divided into femur group (including total femoral, Femoral neck, Ward's triangle, greater trochanter, and intertorch of femur) and spine group (thoracic spine, lumbar spine) according to different sites. Studies that mention both the spine and the femur were excluded. The results showed the serum Mg concentration of postmenopausal women with osteoporosis (BMD of femur) was lower than in healthy controls (SMD = −0.44, 95% CI = −0.77 to −0.12), and BMD of the spine group had the same conclusion (SMD = −0.78, 95% CI = −1.36 to −0.19; Table 2).

We conducted a subgroup analysis based on the number of postmenopausal women with osteoporosis included in this study. The results showed that the serum Mg concentration of postmenopausal women with osteoporosis was lower than that of the normal bone mass group in the studies those included more than 50 postmenopausal women with osteoporosis (SMD = −0.57, 95% CI = −1.04 to −0.11). But in studies that included fewer than 50 postmenopausal osteoporosis, serum magnesium was not associated with osteoporosis (SMD = −0.53, 95% CI = −2.33 to 1.28; Table 2).

The results of the subgroup analysis by age showed that postmenopausal women under the age of 60 with osteoporosis had a lower concentration of serum Mg than the healthy controls (SMD = −0.61, 95% CI = −1.09 to −0.13). However, there was no correlation of serum Mg in postmenopausal women over 60 years old with osteoporosis and the healthy controls (SMD = −0.49, 95% CI = −1.29 to 0.30; Table 2).

### Publication Bias

**Figure 3:**
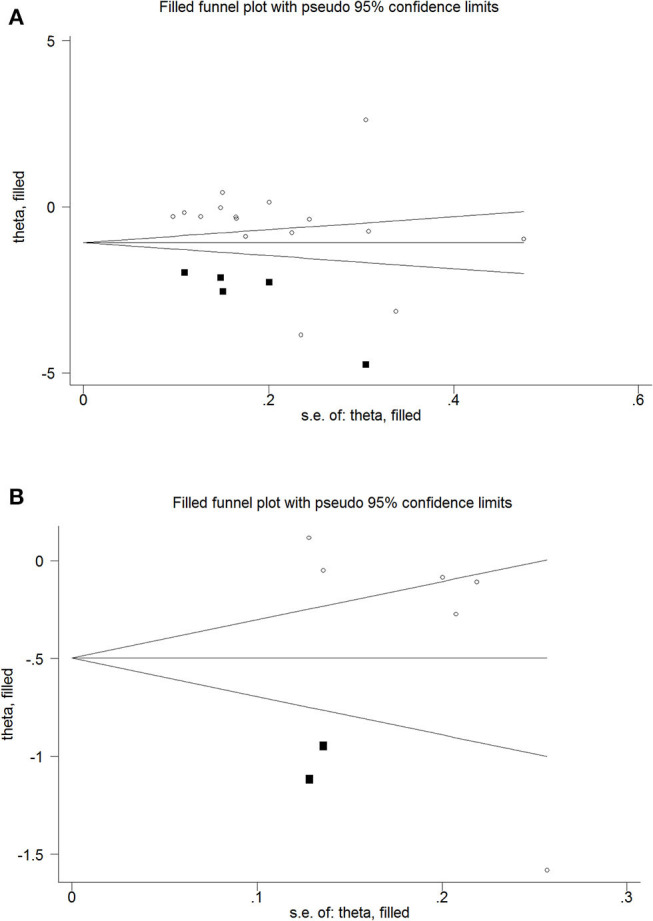
Trim and fill funnel plot for meta-analysis of the association between osteoporosis and healthy controls **(A)**, osteopenia and healthy controls **(B)**.

There was no statistically significant risk of publication bias between women with osteoporosis and healthy controls (Begg's test: *P* = 0.065; Egger's test: *P* = 0.337; Table 3). But the funnel plot was assessed to be asymmetric indicating the possibility of publication bias. To further assess the risk, the trim-and-fill method was used, which showed a statistically significant risk and an undervalued association between the concentration of serum Mg and osteoporosis in previous studies (Estimate = −0.5567, 95% CI = −1.022 to −0.911; Figure 3).

**Table 3:** Publication bias was determined by Begg's test and Egger's regression test.

**Group**	***P* (Begg's test)**	***P* (Egger's test)**
Osteoporosis vs. normal	0.065	0.337
Osteopenia vs. normal	0.024	0.096

However, there was a statistically significant risk of publication bias between women with osteopenia and healthy controls (Begg's test: *P* = 0.024; Egger's test: *P* = 0.096; Table 3). But after applying trim-and-fill, the results were not statistically significant (Estimate = −0.2994, 95% CI = −6.895 to 0.0906; Figure 3).

## Discussion

Our random-effects meta-analysis revealed that postmenopausal women with osteoporosis had a lower concentration of serum Mg than the healthy controls; however, the concentration of serum Mg did not differ between those with osteopenia and healthy controls.

Mg has a close relationship with bone as bone stores comprise about 60% of total Mg. One-third of this skeletal Mg is found in cortical bone and serves as a reservoir for exchangeable Mg, which is beneficial to maintain the extracellular physiological concentrations of cations (9). Accordingly, surface bone Mg increases with loading Mg (32). Mg plays a fundamental role in the biological activation of ATP, which is the main source of energy for the cells (9). Mg deficiency can lead to parathyroid hormone secretion disorders and can affect vitamin D and 1,25(OH)2-vitamin D synthesis. The latter three are important regulators of calcium and bone homeostasis. (10, 33–35). In Mg-deficient animals, newly formed apatite crystals are larger and better structured, which affects the stiffness of the bone (36). Besides, Mg is mitogenic for osteoblasts. Low Mg inhibits osteoblast proliferation by increasing the release of nitric oxide through the up regulation of inducible nitric oxide synthase (37), while it increases the number of osteoclasts by promoting osteoclastogenesis (38). Taken together, maintaining Mg homeostasis can help maintain the homeostasis of osteoblasts and osteoclasts in order to prevent osteoporosis.

The regulation of serum magnesium mainly depends on intestinal absorption and renal excretion (39). In the studies we included, those with diseases and drugs that affect serum Mg levels were excluded. Nevertheless, some chronic underlying diseases, such as renal insufficiency, chronic intestinal disease, or hyperthyroidism, may influence the concentration of serum Mg. This effect cannot be ruled out and may be an important source of heterogeneity in our results. In adults, serum Mg is generally independent of age and sex. The increase of serum Mg was only slightly higher in those very elderly women who were measured after intense short-lived active or vegetarian (39). In general, Mg deficiency is mainly due to lower consumption, inadequate absorption, and/or increased excretion (40). Although some possible confounders were controlled by inclusion criteria, dietary intake of Mg may have a significant effect on serum Mg. Considering that the diet structure will not change much in the short term within a region, we conducted a subgroup analysis based on the regional category. Our results showed a lower concentration of serum Mg was found in European postmenopausal women with osteoporosis than in healthy controls. This may be related to the insufficient intake of Mg in the European diet. While we analyzed the concentration of serum Mg between women with osteopenia and healthy controls, no correlation was found.

In order to further explore the heterogeneity between studies, we conducted subgroup analysis based on the sites of BMD, sample size, and age. Results showed postmenopausal women with osteoporosis had a lower concentration of serum Mg than the healthy controls in those studies which had more than 50 postmenopausal women with osteoporosis. This means that more studies with large sample sizes may prove the correctness of our conclusions. Another subgroup analysis conducted by sample size showed serum Mg levels were lower in osteoporosis than in the normal group, regardless of whether the femur or the spine were measured. A meta-analysis reported there were no significant correlations observed between Mg intake and BMD in the lumber spine (41). In general, the main source of Mg is daily intake, Mg dietary deficiency can cause a decrease in the concentration of serum Mg and a loss of systemic bone mass (42). So, the correlations observed of BMD in spine with serum Mg and Mg intake were difference. This may be related to the transformation and storage of Mg. We also tried to do a subgroup analysis by age, there was no correlations of serum Mg in postmenopausal women over 60 years old with osteoporosis and the healthy controls, postmenopausal women under the age of 60 with osteoporosis had a lower concentration of serum Mg than the healthy controls.

Our results showed that postmenopausal women with osteoporosis had a lower concentration of serum Mg than healthy controls but found no significant correlation between osteopenia and healthy controls. Although we combined the concentration of serum Mg between postmenopausal women with osteopenia and healthy controls and attempted to perform subgroup analysis by age and region, the results were inconclusive.

Serum Mg concentration is a useful and easily measured measure of magnesium in bone. However, in chronic latent magnesium deficiency, blood magnesium levels may remain normal despite significant reductions in tissue and bone magnesium levels. Using blood magnesium levels to determine total magnesium levels may underestimate magnesium deficiency in healthy and ill people (40). This is consistent with the underestimation of the relationship between serum Mg concentration and osteoporosis that we obtained by building a trim-and-fill funnel plot. Serum Mg levels are only measured once, its correlation with osteoporosis after menopause just reflects the relationship between the two at the time, will not be able to accurately evaluate the extent of the past or the future osteoporosis. But this does not mean that the study of serum magnesium is meaningless. Through linear regression analysis, Akizawa et al. (43) calculated that the correlation coefficient between Japanese daily Mg intake and serum Mg concentration was 0.29 and suggested to calculate the Mg intake through serum Mg concentration to ensure the optimal individual serum magnesium concentration. By adjusting the Mg intake, the optimal serum Mg level in the human body is maintained to achieve the purpose of preventing related diseases (43). Besides, our meta-analysis found the relationship between serum magnesium levels and post-osteoporosis, providing a basis for future prospective studies to verify the relationship between serum magnesium levels and osteoporosis or BMD.

### Strengths and Limitations

To our knowledge, this is the first meta-analysis to explore the relationship between serum magnesium in postmenopausal women with osteoporosis, osteopenia, and normal. In the studies selected for the meta-analysis, 2,776 postmenopausal women are represented. We performed a subgroup analysis based on region, sites of BMD and sample size.

However, some limitations of our study must be considered. Firstly, the sample sizes of these studies varied significantly, which would increase heterogeneity. In contrast, the women in the included studies could be from the same place or hospital resulting in reduced heterogeneity. In order to measure heterogeneity, we conducted subgroup analysis and found that region, the sample sizes and age may be important reasons for the high heterogeneity of our studies. However, we cannot fully eliminate heterogeneity because of the diversity of studies. Secondly, only two studies mentioned adjustment of confounding variables, like age, body mass index, etc. (25, 29). This makes it difficult to exclude the influence of other confounders in the study which may have resulted in a bias in our analysis. Additionally, there are so few studies looking at the relationship between serum Mg and osteopenia that the amount of data we collected is very limited and our study may be underpowered. Finally, the studies that we included could be influenced by demographic characteristics, serological limitations, and other factors. Because of these reasons, we recommend that our conclusions are viewed conservatively.

## Data Availability Statement

Publicly available datasets were analyzed in this study. This data can be found in PubMed and CnKI.

## Author Contributions

BY and DY designed the meta-analysis. DY, JJ, and NW performed the literature retrieval and the data extraction. JC and SY contributed the article writing. All authors read and approved the final manuscript.

## Conflict of Interest

The authors declare that the research was conducted in the absence of any commercial or financial relationships that could be construed as a potential conflict of interest.
